# Guidelines for Assessing and Enhancing the Organizational Vitality of Pharmacy Educational Programs: A Call to Action!

**DOI:** 10.3390/pharmacy10050128

**Published:** 2022-10-08

**Authors:** Ashim Malhotra, Jeremy Hughes, David G. Fuentes

**Affiliations:** 1 Department of Pharmaceutical and Biomedical Sciences, California Northstate University College of Pharmacy, 9700 West Taron Drive, Elk Grove, CA 95757, USA; 2 College of Pharmacy, Chicago State University, 9501 S. King Drive, Chicago, IL 60628, USA; 3 School of Nursing and Health Innovations, University of Portland, 5000 N. Willamette Blvd, Portland, OR 97203, USA

**Keywords:** organizational vitality, knowledge capital, human capital, intellectual capital, market value

## Abstract

Organizational vitality encompasses organizational mission and identity, organizational purpose and values, and employee engagement, cohesiveness, anxiety, and information sharing. Using the organizational vitality framework consisting of the following five pillars: (1) human, (2) knowledge, (3) intellectual, (4) financial capital, and (5) market value, we propose a reflection guide and specific calls to action for academic leaders including deans, department chairs, assistant/associate deans, and others within pharmacy and healthcare education systems. Our overall aim is to provide a blueprint for academic leaders to assess and enhance the organizational health, vitality, resiliency, and sustainability of their pharmacy educational programs using an established organizational vitality framework. This guide can help academic leaders at all levels to reflect on their organization’s vitality and use the steps outlined here to renew conversations about faculty life, identities as leaders, the global pharmacy Academy’s core mission and values, and the pursuit of work-life harmony in the context of their pharmacy schools’ organizational vitality. All leaders within pharmacy educational programs should identify and embrace a holistic and guided framework that emphasizes organizational vitality.

## 1. Introduction

Operationally, pharmacy schools and colleges in the United States exist as independent organizations with autonomy in structural, financial, procedural, human resources, and management operations within the university framework. Like other organizations, pharmacy programs find themselves amid a rapidly transforming cultural, legal, market, and sociopolitical ecosystem [1,2]. While remaining true to the organizational core mission, vision, and values, the success of each pharmacy program is undeniably linked to its ability to effectively manage the operational, community, and personnel costs while building a resilient and adaptive organization [2,3,4]. Pharmacy educational programs may benefit from using the guided organizational vitality model to create a systematic and directional approach to ensuring they add value as an organization. As new and existing academic leaders take the helm of organizations, it is important to centralize a holistic framework that emphasizes organizational vitality. Supervisors and managers, including those in academia, are being asked to take on more roles than ever before such as serving as coaches, advisors, mentors, and sponsors. Additionally, many leaders must acclimate to the culture of organizations. There is a need to consider the human capital that serves as the lifeblood of the organization, emphasize mission-based and values-based faculty and staff recruitment, professional development, succession planning, clarity in roles and responsibilities, and employee well-being. A paradigm shift is needed from engaging in business-as-usual, sometimes focused only on the curricular outcomes, to giving primary attention to organizational health, employee wellness, and engagement [5,6]. Acknowledging the associated complexities of structures and principles at work within pharmacy education will foster systems thinking about holistic organizational vitality, leading to the sustainability of programs during challenging times. 

The COVID-19 pandemic has negatively impacted all organizational constituents of pharmacy educational programs such as human, financial, intellectual, and knowledge capital. This has been amplified by decreased admission applications, financially strained operations, and burdened strategic plans [7,8,9,10,11,12,13,14]. These challenges worsened faculty stress, tested resilience, and produced a level of lasting anxiety, all contributing to burnout and high levels of faculty turnover [7,8,9]. 

**Figure 1:**
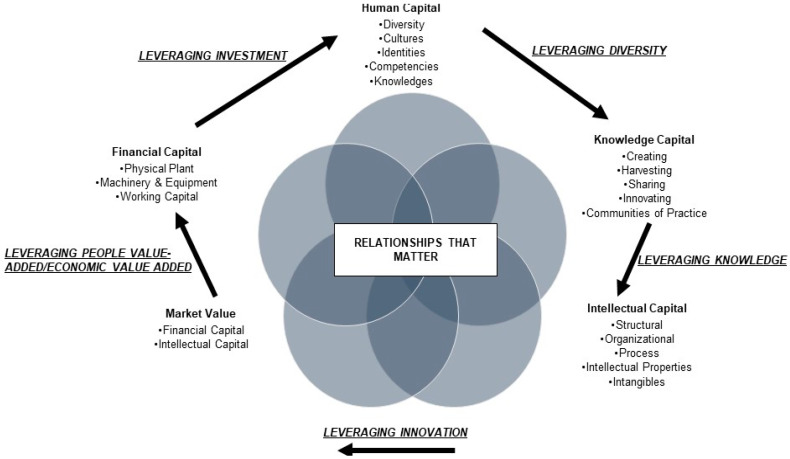
The Organizational Vitality Pillars Framework. Based on [17].

As organizations, pharmacy programs adhere to their core mission, vision, and values, while effectively managing to adapt to challenges and building capacity for resiliency, adaptation, and sustainability [3,4,15,16,17]. Leaders at all levels within the Academy can leverage the organizational vitality framework to create healthy and sustainable programs that can be measured using established tools on employee engagement and industry success metrics [15,16,17]. The five focus areas of the organizational vitality framework combine concepts of organizational development and knowledge management [17]. The framework is holistic and can be applied to all stakeholders within the pharmacy Academy, making it a strong and compelling vision for leaders to use and reflect on, ensuring that they are providing support to all stakeholder groups in the organization with equity [17]. Thus, the organizational vitality framework is an efficient way to address stakeholder needs and promote organizational health. As depicted in Figure 1, this organizational vitality framework identifies five essential components of an organization: (1) human, (2) knowledge, (3) intellectual, (4) financial capital, and (5) market value [15,16,17]. 

The organizational vitality framework has several interconnected and non-linear pillars. The elements of human capital, and their unique attributes, connect with how knowledge capital is operationalized. Intellectual capital and financial capital are related to the organization’s needs and how they leverage human capital. Market value is determined heavily by the combination of financial capital and intellectual capital. All these interconnected pillars are further facilitated by productive, civil, and collegial relationships that underpin organizational health. Table 1 depicts example questions to consider for each of these five pillars. 

As an example of how the organizational vitality framework is centered around relationships, if the five pillars of knowledge capital, intellectual capital, human capital, financial capital, and market value are not integrated, the impact of industry and technology changes, impacting employee attrition and resulting in unfulfilled customer demands, conflicting priorities, insurmountable workload, and inefficient team processes that can sink an enterprise [1,2,3,4,15,16,17]. When combined, these various threats can result in heightened employee anxiety and a cyclical exacerbation of the challenges described. A vicious cycle can spiral the organization downwards and some organizations may never fully recover if these key elements are ignored. 

Here, we suggest the universal adoption of this organizational framework for all schools and colleges of pharmacy in the United States. Additionally, while there is significant variability in the nature, mission, and scope of pharmacy education programs across the globe, the adoption of this idea, particularly the careful and deliberate integration of the following five pillars of this framework will help all pharmacy schools, regardless of the structure, to achieve organizational efficiency and vitality. 

## 2. Components of the Organizational Vitality Framework

### 2.1. Human Capital

Human capital can be argued to be one of the most important aspects of this framework. Leaders often believe they are responsible for the results, but what if they focused on their employees instead? This shift would allow employees to fully focus on the results of the organization. Human capital includes diversity, culture, identity, competency, and knowledge [17]. Supervisors across the Academy must adopt strategies to promote appreciation for their colleagues at all levels. This can directly facilitate belonging, connection, and psychological safety [18]. The element of appreciation requires that employees be seen for attributes, including both strengths and weaknesses, and elements of diversity, culture, and identity [19]. 

#### Applying the “Human Capital” Element to Pharmacy School Operation

Organizations can achieve this by proactively recruiting and retaining diverse students, faculty, and preceptors, normalizing diverse, equitable, inclusive, and antiracist (DEIA) discourse, and promoting inclusive strategic planning and commitment to respect and civility. Furthermore, the elements of competency and knowledge can progress from exposure to new experiences to their application, increased self-efficacy, decentralized decision making, and increased leadership capacity at all levels [20]. Program deans can work closely with their university administration and human resources to guide searches; assistant and associate deans can develop operational strategies aimed at student admissions, support services, and progression, and department chairs can adopt coaching mindsets to ensure the inclusion of departmental talent. Another example of investing in human capital may include sending a faculty member to the AACP Institute themed on curricular revitalization, then having them chair the curriculum revision when returning to the program. 

### 2.2. Knowledge Capital

If human capital is stabilized, the knowledge capital pillar has a greater chance of developing. Knowledge capital encompasses creating, sharing, and harvesting information that is created collectively from diverse perspectives [17]. Supervisors and leaders at all levels of the pharmacy academy can boost organizational vitality by integrating formal knowledge management systems, promoting communities of learning and practice, enabling cross-unit collaborations, encouraging sharing of resources, and demolishing siloes [1,2,3,4]. Placing energy into these areas can catalyze communications among diverse groups, promote expertise sharing, and adopt courage in the face of risk-taking. 

#### Applying The Organizational Framework of “Knowledge Capital” to Pharmacy Schools

Program deans can achieve this by leading their teams to amass and curate relevant knowledge capital from a variety of sources. Assistant/associate deans, department chairs, and directors at all levels possess crucial information from operations, programmatic assessment, and quality control parameters such as surveys, meetings, projects, strategic initiatives, town halls, and other informal and formal feedback mechanisms. Academic leaders can create a centralized repository for data and provide support to manage it, hiring personnel to support knowledge management or assigning this to key individuals with a systems mindset. Setting norms on knowledge management practices may add to the vitality and sustainability of the program. While technology integration is often envisioned as the solution for knowledge management, added technology may not be necessary, and formal systems and personnel can help manage and curate institutional memory and formal knowledge management into knowledge capital.

Additionally, program deans, administrators, and others can reach out to colleagues and develop communities of learning and mastermind groups across organizations. Such efforts offer mechanisms for creating collaborations, integrating diverse ideas, and developing communities of support, thus creating new and shared organizational knowledge capital. See Table 1 for examples. 

### 2.3. Intellectual Capital

Intellectual capital encompasses structural components in the organizational vitality framework which provide safety, boundaries, and processes that employees at all levels need to promote clarity [17]. Using both leadership and management, organizations can set the vision with leadership while addressing the safety and structure achieved through managing clear policies, practices, and operations. While not often seen as the most exciting components of an organization, the structural policies and procedures inherent to management are essential to providing clarity, minimizing organizational risk, and promoting the value of transferable knowledge that can withstand personnel changes [3,4].

#### Incorporating the “Intellectual Capital” Framework in Pharmacy Schools

Deans and executive teams lead the development of mission-based and values-focused policies that clearly outline critical organizational structures and processes. Meeting with diverse stakeholders to hold policy summits and discussions can highlight how policies are connected to the organizational mission and the role of individuals and committees for positive change. 

### 2.4. Financial Capital

While many elements of academic institutions make them unique from other industries, pharmacy educational programs must operate to promote stability and sustainability, recognizing that financial capital is critical for organizational health [17]. Physical space, equipment for research and operations, and working capital are necessary components needed to develop sustainable budgets, connect financial resources to the strategic planning for the future, to move initiatives that spark growth into new markets [1,2,3,4,15,16,17]. With such expansion, it would be possible to attract different student types and magnetize the recruitment of diverse faculty and staff towards promoting greater inclusivity within the Academy [11,13,14,15]. Similarly, diversifying academic portfolios to include multiple types of degree and certification programs, such as stackable credentials within the pharmacy curriculum and related certificates and Masters and Doctoral programs of study, can draw in different student populations. 

#### Applying the “Financial Capital” Element to Pharmacy School Organization

Program deans and members of executive teams can educate existing stakeholders on the various revenue streams that programs provide to the larger university budget. Conversations about operations costs can be shared to promote both transparency and professional development on the aspects necessary to leverage resources and run a pharmacy program. Faculty, staff, and students can benefit from a clearer window into the financial health of programs and may appreciate having a broader understanding of organizational finances. This can also be achieved without needing to provide confidential data or sharing in excess. Understanding the current financial state of the organization can be helpful for faculty, staff, and students to improve stewardship and how ideas for new revenue streams can be best utilized. 

### 2.5. Market Value

Within the organizational vitality model, both the financial and intellectual pillars intersect to inform and establish market value [17]. Leaders need to leverage the expertise within their teams and academic communities to develop a coherent and powerful narrative communicating their value proposition (Table 1). 

#### Building The “Market Value” Component to Promote Pharmacy School Vitality

It has become necessary to develop value structures that resonate with increasingly diverse prospective students, families, and other stakeholders [3,4]. The elements of each organization’s value proposition are intimately connected to its core values, mission, identity, and culture, further demonstrating the interweaving of all elements presented in the organizational vitality framework [17]. To arrive at this value proposition in an engaged and enlightened way, leaders must ask powerful questions, listen more than they speak, and create an environment of belonging and psychological safety to ensure their team members at all levels can be willing and able to share their valuable ideas and perspectives [1,4]. Only through a collective process can a solid value proposition that accurately describes the organization succeed and grow. 

## 3. Conclusions

Our work in challenging the academy to adopt Adkins and Vicenzi’s organizational vitality framework is novel in the following ways. As depicted in Figure 1, each step must be sequentially adopted. Once deployed, there is a need to periodically monitor progress after all the steps have been implemented. Additionally, the framework works best through inclusivity. For example, while some schools of pharmacy may already excel with specific components of the framework such as budget management and finance, these are typically placed in the Dean’s office. However, the model calls for inclusive sharing and planning of all components throughout the organizational structure. While some pharmacy schools may be gainfully employing components of the model, it is important to emphasize that the framework works best if all five pillars are planned for and enacted sequentially.

As schools of pharmacy change in response to external market needs and threats, internal faculty and staff resources, and the larger educational environment, there is an urgent need to effectively manage the internal organizational environment of human, financial, intellectual, and knowledge capital and market value [1,2,3,4,16,17]. Adopting a flexible organizational vitality model and mindset will foster sustainability, growth, and well-being in all five organizational areas.

## Figures and Tables

**Table 1:** Considerations to Guide Reflection on Organizational Vitality for Pharmacy Schools.

Human Capital	Do Academic Leaders…? Establish clear cultural missions and values that inform searches for faculty, staff, administrators, and students. Integrate curricula on the multiple identities and expectations of the roles of healthcare professionals. Crystallize educational outcomes and competencies towards progression and academic growth. Capture the linked elements of knowledge necessary for establishing purpose, culture, norms, and desired values at the organizational level. Leverage relationships and connections to create internal and external partnerships, mastermind groups, and communities of practice and learning. Promote structures for talent development and growth opportunities connected to stretch projects and succession planning. Inculcate a culture of, and training for, a coaching mindset and relationship between supervisors and their direct reports. Define, implement, and reward individuals for collegial, civil, and curious exchanges that further the tripartite mission productively. Set the environment to engage in conversations leading to discourse conducive to innovative behaviors in a culture tolerant of acceptable risk, psychological safety, and belonging. Balance flexibility towards employees regarding virtual and in-person work with careful consideration for the impact of workforce fragmentation.
Knowledge Capital	Create formal infrastructures for handling, managing, and sharing information across the organization, and with external partners as appropriate. Align existing information with organizational values, purpose, and the evolving story of the organization is useful to communicate the shared mission with current and future partners. Identify and manage silos causing information to become “lost” or used in ways that cannot serve the entire organization. Appoint or acquire personnel to oversee the process of knowledge management. Develop rules and norms for knowledge management in alignment with the organization’s needs and applicable regulations.
Information Capital	Outline the most critical, and values-based, organizational structures and processes. Meet with diverse stakeholders to hold policy summits and discussions to educate on policy development. Use evidence-based reasons for policy changes as organizations evolve. Encourage, facilitate, and systematize sharing of ideas generated within the organization, and with external organizations.
Financial Capital	Work with their existing stakeholders and provide education on the various revenue streams informing the university budget. Hold conversations about the myriad costs of operations. Promote both transparency and professional development on the aspects necessary to leverage pharmacy program resources.

Based on [17].

## Data Availability

Not applicable.
